# Temporal Predictions in Space: Isochronous Rhythms Promote Forward Projections of the Body

**DOI:** 10.3389/fpsyg.2022.832322

**Published:** 2022-05-04

**Authors:** Laura Ferreri, Rémy Versace, Camille Victor, Gaën Plancher

**Affiliations:** Laboratoire d’Étude des Mécanismes Cognitifs, Université Lumière Lyon 2, Lyon, France

**Keywords:** temporal prediction, isochronous rhythm, VAAST, body projection, embodied cognition

## Abstract

A regular rhythmic stimulation increases people’s ability to anticipate future events in time and to move their body in space. Temporal concepts are usually prescribed to spatial locations through a past-behind and future-ahead mapping. In this study, we tested the hypothesis that a regular rhythmic stimulation could promote the forward-body (i.e., toward the future) projections in the peri-personal space. In a Visual Approach/Avoidance by the Self Task (VAAST), participants (*N* = 24) observed a visual scene on the screen (i.e., a music studio with a metronome in the middle). They were exposed to 3 s of auditory isochronous or non-isochronous rhythms, after which they were asked to make as quickly as possible a perceptual judgment on the visual scene (i.e., whether the metronome pendulum was pointing to the right or left). The responses could trigger a forward or backward visual flow, i.e., approaching or moving them away from the scene. Results showed a significant interaction between the rhythmic stimulation and the movement projections (*p* < 0.001): participants were faster for responses triggering forward-body projections (but not backward-body projections) after the exposure to isochronous (but not non-isochronous) rhythm. By highlighting the strong link between isochronous rhythms and forward-body projections, these findings support the idea that temporal predictions driven by a regular auditory stimulation are grounded in a perception-action system integrating temporal and spatial information.

## Introduction

Music is a dynamic process occurring over time. During music listening, we constantly generate hypotheses about what could happen next in terms of time (i.e., *when* expectations) and content (i.e., *what* expectations; Tillmann et al., 2014). While *what* expectations are usually manipulated through changes in melodic or harmonic sequences (i.e., the spectral structure), *when* predictions are mainly related to the temporal structure of music, based on repeated beats (Koelsch et al., 2019). When exposed to isochronous rhythms (i.e., auditory stimulations with temporally equidistant beats), listeners are able to implicitly predict where each beat will fall in time (Cannon and Patel, 2021). At a behavioral level, temporal predictions driven by periodic stimulation have been shown to improve performance by increasing perceptual sensitivity and reducing response latencies (e.g., Jones et al., 2002; Stefanics et al., 2010; Lawrance et al., 2014; Morillon et al., 2016).

Human ability to perceive beat is closely related to movement on both behavioral and neural levels. People show a spontaneous tendency to move (from finger tapping to entire body movement) in response to the beat of rhythmic sound (Repp, 2005; Damm et al., 2020). In particular, successful motor synchronization seems to be driven by the more regular and predictable musical sequences (Dalla Bella et al., 2013). Furthermore, brain studies highlighted that mere listening to musical beat (i.e., in the absence of movement) can activate regions of the motor system (Zatorre et al., 2007; Grahn and Rowe, 2009). Even simple human actions such as walking are coordinated and rhythmic and have been shown to operate at a spontaneous preferred tempo of ∼1.5–2 Hz (see e.g., MacDougall and Moore, 2005; Zalta et al., 2020). Moving to the beat has been shown to benefit auditory perception tasks as well (Manning and Schutz, 2013; Morillon et al., 2014). In this case, the motor system, by simulating the rhythmic actions, might provide for temporal predictions about beat times in the auditory regions (Patel and Iversen, 2014; see also Gatti et al., 2021).

Taken together, these studies suggest that a regular rhythmic stimulation, by tapping on perceptual and motor processes, promotes people’s body movement in space. A regular auditory stimulation is likely to stimulate people’s ability to anticipate future auditory events in time. Indeed, according to the dynamic attending theory (Jones, 1976; Jones and Boltz, 1989; Large and Jones, 1999), the attentional resources are not distributed continuously and equally but cyclically develop over time. By entraining internal oscillations to an external regular rhythm, temporal regularities can then guide attention. Consequently, perceivers can develop predictions about the temporal occurrence of a future event and allocate more attentional resources at the expected moment, and this in turn results in enhanced cognitive processing of an event occurring at this moment (see e.g., Jones et al., 2002; Escoffier et al., 2010; Fanuel et al., 2018).

Such time–space link becomes particularly interesting within the embodied cognition framework (Glenberg, 1997; Wilson, 2002; Barsalou, 2008; Versace et al., 2014), which considers the abstract concept of time as embedded in a perception-action system and integrated with spatial information (Lakoff and Johnson, 1980; Núñez and Cooperrider, 2013). In daily life, we tend to prescribe temporal concepts to spatial locations through a past-behind and future-ahead mapping (e.g., we *move back to the past*, and *look forward to the future*; Boroditsky, 2000). Several studies highlighted that the ability to anticipate future events or mentally travel through the past is consistently related to physically moving in space forward or backward, respectively (i.e., a front-back representation; Torralbo et al., 2006; Miles et al., 2010; Rinaldi et al., 2016; Aksentijevic et al., 2019). For example, in a word categorization task, Rinaldi et al. (2016) showed that participants were faster to step forward in response to future-than past-related words, whereas they stepped backward faster in response to past-than to future-related words.

By showing a link between temporal processes and space-motor programming employing mental time traveling paradigms (i.e., related to remembering past events or thinking about the future; Tulving, 2002), these studies support the idea that cognition is dynamically rooted in the interactions that the body maintains with the environment over time (Barsalou, 2008; Bender and Beller, 2014; Versace et al., 2014). Although the tight coupling between the temporal qualities of music and movement is well stated in literature (see Levitin et al., 2018), to the very best of our knowledge, no study so far manipulated the predictability of a rhythmic stimulation to study the link between the abstract concept of time, spatial references frames, and body movements. Therefore, we aim here at investigating whether the ability to predict future events driven by a regular auditory stimulation can specifically influence movements in space through a backward–forward mapping.

To this aim, we employed an adapted version of the Visual Approach/Avoidance by the Self Task (VAAST, Rougier et al., 2018) to test participants’ implicit forward and backward body movements following regular (i.e., isochronous) or irregular (non-isochronous) auditory rhythmic stimulations presented at different tempi. This task has been effectively used in previous studies to show the approach/avoidance compatibility effect (Solarz, 1960). Typically, participants provide faster responses to approach positive (vs. negative) stimuli and to avoid negative (vs. positive) stimuli presented in a virtual scene. Here, we used it to study whether the temporal regularity of auditory stimuli could impact the time taken to approach a visual scene or avoid it. We asked participants to make as quickly as possible a perceptual judgment on the visual scene (i.e., whether the metronome pendulum represented on the scene was pointing to the right or left). The responses could trigger a forward or backward visual flow, respectively approaching or moving them away (i.e., avoidance) from the scene.

We made the hypothesis that if temporal predictions promoted by a regular (but not irregular) rhythmic stimulations are grounded in a perception-action system integrating temporal and spatial information, and therefore are tightly related to forward body movements, then an isochronous rhythm should lead to faster responses associated to approach (i.e., forward) behavior than a less predictable, non-isochronous rhythms.

## Materials and Methods

### Participants

Twenty-four non-musician participants (17f, mean age = 22.08, *SD* = 3.19), with normal or corrected-to-normal vision and reported normal audition were included in the study. Statistical sample size analysis run on G*power, based on an effect size of 0.25 and 95% confidence interval for the effect for a 2 × 2 × 2 within subject design (see Analysis section) indicated a total sample size of 23. All participants signed a written informed consent before the participation. The study was conducted in accordance with the Helsinki Declaration, seventh revision. Ethical review and approval were not required for the study on human participants in accordance with the local legislation and institutional requirements.

### Auditory Stimuli

**FIGURE 1:**
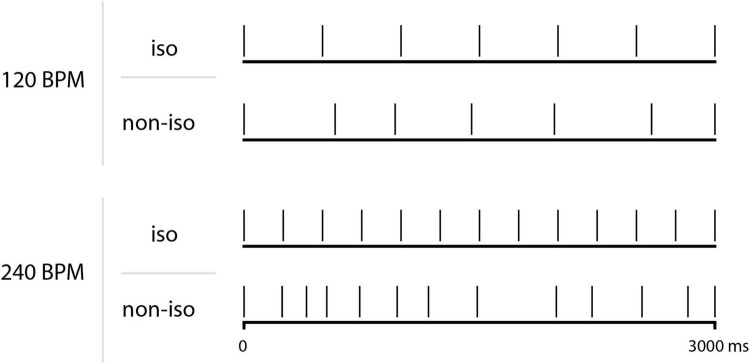
Schematic representations of one isochronous (iso) and one non-isochronous (non-iso) auditory sequence, for each tempo (i.e., 120 and 240 BPM). Each vertical line represents a tone (see Table 1 for a complete description).

Auditory stimuli consisted of 3-s (enough to entrain synchronization, Trapp et al., 2020) isochronous and non-isochronous bass drum-like rhythmic tone sequences. To control whether an isochronous-effect over body movements could be generalized to different frequencies rather than being limited to preferred natural cadences (see Zalta et al., 2020), the auditory stimuli were presented at two different tempi, namely 120 (i.e., 2 Hz, similar to the preferred natural frequency) and 240 BPM (i.e., 4 Hz). In particular, for the isochronous rhythms (2 in total), the tone was regularly presented at a tempo of 120 or 240 BPM. The non-isochronous rhythms sequences (12 in total) consisted of the same tone presented for 6 or 12 subsequent pulsations with random intervals. The onsets of the first and the last tones remained constant across sequences (i.e., 0 and 3,000 ms, respectively; Figure 1 and Table 1).

**TABLE 1:** Description of onsets (in ms) in isochronous (iso) and non-isochronous (non-iso) tones sequences, for the 120 (a., i.e., a total of 7 tones) and 240 BPM tempi conditions (b., i.e., a total of 13 tones).

a.	Tempo	120 BPM												
	Tones sequence	1	2		3		4		5		6		7	
	Iso	0	500		1000		1,500		2000		2500		3000	
	Non-iso1	0	676		997		1486		1988		2185		3000	
	Non-iso2	0	805		1244		2117		2309		2618		3000	
	Non-iso3	0	245		434		870		1622		2683		3000	
	Non-iso4	0	434		1185		2247		2686		2818		3000	
	Non-iso5	0	248		436		1945		2247		2813		3000	
	Non-iso6	0	186		997		1185		1624		1999		3000	

**b.**	**Tempo**	**240 BPM**												
	**Tones sequence**	**1**	**2**	**3**	**4**	**5**	**6**	**7**	**8**	**9**	**10**	**11**	**12**	**13**

	Iso	0	250	500	750	1000	1250	1500	1750	2000	2250	2500	2750	3000
	Non-iso1	0	246	374	502	746	997	1125	1499	1996	2248	2562	2867	300
	Non-iso2	0	246	617	748	877	1182	1314	1439	1745	1999	2433	2564	3000
	Non-iso3	0	249	500	625	997	1245	1497	1628	1999	2248	2496	2624	3000
	Non-iso4	0	251	371	746	874	1000	1248	1497	1622	1996	2125	2253	3000
	Non-iso5	0	235	374	746	997	1248	1376	1499	1622	2125	2250	2559	3000
	Non-iso6	0	245	498	873	1000	1248	1498	1749	1873	2121	2248	2562	3000

*The onsets of the first and the last tones remained constant across sequences.*

In order to control for possible affective responses associated to the auditory stimuli, participants’ ratings of arousal and valence/pleasantness were collected after the main experimental task (see section below). After each auditory sequence, participants had to indicate, by moving a cursor on a bar on the screen (values ranging from 0 to 100), how much they rated the stimulus arousing (from very relaxing to very exciting) and enjoyable (i.e., valence, from lowly enjoyable to highly enjoyable).

### Visual Approach/Avoidance by the Self-Task Task

The Visual Approach/Avoidance by the Self-Task (VAAST) developed by Rougier et al. (2018) relied on sensorimotor indices provided by a realistic visual flow on the screen stimulating whole-body movements in relation to an emotional stimulus (i.e., target words). In a series of six experiments, the authors showed that participants provided faster response times to approach (via a zoom-in on the screen approaching the subject to the scene) positive stimuli and avoid (via a zoom-out moving the subject away from the scene) negative stimuli, than the reverse (also known as the approach/avoidance compatibility effect, Solarz, 1960).

**FIGURE 2:**
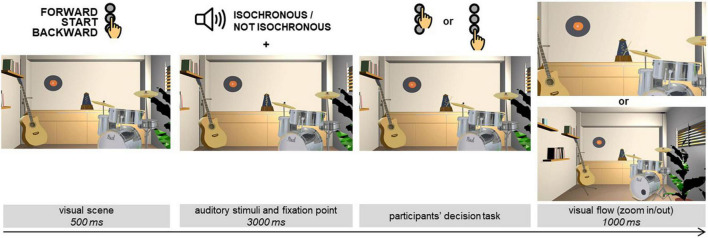
Schematic representation of the experimental procedure.

While the procedure originally investigated virtual body movements in relation to emotional stimuli, here we employed it to study whether the temporal regularity (or irregularity) of auditory stimuli could impact the time taken to produce a motor response generating forward (i.e., approaching) or backward (i.e., avoiding) visual flow. In our study, participants were seated in a comfortable chair in a soundproof experimental room and wore headphones. During a training phase, they were instructed to use with the index finger of the dominant hand three adjacent buttons disposed vertically on the keyboard (see Figure 2): a middle “start” button to start each trial, and two external buttons to perform the categorization task labeled “forward” (triggering the approach) and “backward” (triggering the avoidance).

The VAAST task consisted of 120 trials: 60 presented in the isochronous and 60 in the non-isochronous condition (i.e., 30 for the 120 BPM condition, and 30 for the 240 BPM one). The trials were divided into 5 blocks of 24 trials each (i.e., 6 trials for each experimental condition for each block). For each trial, a visual scene was presented on the screen. The visual scene represented a music studio with a metronome in the middle (see Figure 2). This scene was constructed in order to be realistic and coherent with the presented auditory stimulation. Participants were instructed to fixate a white circle placed in the middle of the screen, below the needle of the metronome. As soon as participants pressed the “start” button, the 3-s auditory stimulation (i.e., isochronous or non-isochronous, in a random order) started. During the auditory stimulation, the needle of the metronome was stationary. Immediately after, the needle of the pendulum in the middle of the visual scene randomly pointed to the left or to the right. Therefore, there was no pause between the end of the auditory stimulation and the moving needle on the screen. Participants were asked to indicate as fast and as correctly as possible whether the pendulum of the metronome was pointing to the right or left by pressing on the “forward” or “backward” button. The association between forward/backward button and left/right response was counterbalanced across participants. For correct responses, according to the participants’ approach/avoidance action, the visual environment was zoomed in (i.e., approach, “forward” button) or zoomed out (i.e., avoidance, “backward” button) by 30% after each button press, thus giving the visual impression to move forward or backward as a consequence of these actions. After the zoom, the initial scene was reset on the screen for the next trial. The incorrect answers did not trigger any visual flow and were accompanied by a visual feedback of error (i.e., a red cross on black background).

### Analysis

Reaction times (RTs) of correct responses to the perceptual categorization task were first computed for each participant. Outlier RT values (i.e., ±2.5 standard deviations from the mean) within each experimental condition, corresponding to the 5.49% of the data, have been excluded from the analyses. In order to test the results of the VAAST task, we run a repeated-measures ANOVA (JASP 0.11.1.0) with correct responses RTs as a dependent variable and body movement (approach vs. avoidance), rhythm (i.e., isochronous vs. non-isochronous) and tempo (i.e., 120 vs. 240 BMP) as within-subject factors. In an additional analysis, in order to test for an eventual congruency effect between left-right responses and past-future representations, we added the left/right counterbalancing (i.e., the association between forward/backward button and left/right response across participants) as between-subject factor.

In order to investigate possible difference in subjective ratings of auditory stimulation, we run repeated-measures ANOVAs with rhythm (i.e., isochronous vs. non-isochronous) and tempo (i.e., 120 vs. 240 BMP) as within-subject factors on both arousal and valence subjective ratings.

## Results

**FIGURE 3:**
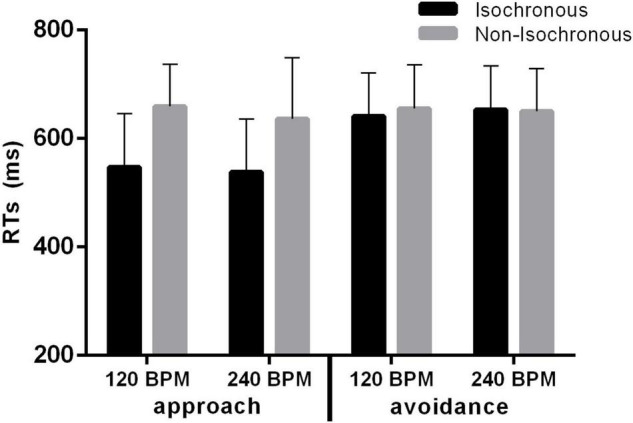
Results of the VAAST task showing RTs for correct responses according to the body movement (approach/avoidance), auditory rhythmic stimulation (isochronous/non-isocrhonous) and tempo (120/240 BPM). Analysis revealed significant main effects of body movement and rhythm, as well as a significant interaction body movement × rhythm, thus indicating that participants were faster in approach (vs. avoidance) response following an isochronous (vs. non-isochronous) auditory stimulation.

RTs analysis showed a good overall performance of participants, with the 94.51% of correct responses in the left-right categorization task. Repeated-measures ANOVA on VAAST task revealed an effect of body movement, indicating that participants were faster in providing responses associated to approach rather than avoidance movements [*F*(1, 23) = 16.53 *p* < 0.001, η^2^ = 0.135; Figure 3]. A main effect of rhythm was also observed, with faster responses following isochronous than non-isochronous auditory stimulation [*F*(1, 23) = 25.77, *p* < 0.001, η^2^ = 0.145], and independently from the tempo variations (*F* < 1). Crucially, results showed a significant interaction between body movements and rhythm [*F*(1, 23) = 57.79, *p* < 0.001, η^2^ = 0.114], with participants providing faster approaching responses after isochronous than after non-isochronous auditory stimulations (*t* = –8.139, *p* < 0.001, Bonferroni-corrected), and without significant difference between rhythmic conditions in the avoidance body movement responses (*t* = –0.49, *p* = 1.000, Bonferroni-corrected). We found no significant effect of counterbalancing of forward/backward movement and right/left responses [*F*(1, 22) = 1.71, *p* = 0.204, η^2^ = 0.034], nor any interactions involving this factor.

The repeated-measures ANOVAs on affective ratings did not show any significant effect of rhythm and tempo on arousal (*F* < 1 for both rhythm and tempo) nor valence subjective ratings [for rhythm, *F* < 1; for tempo, *F*(1, 23) = 1.140, *p* = 0.297; see Table 2].

**TABLE 2:** Means and standard deviations (SD) of mean arousal and valence subjective ratings according to auditory stimulation (Iso, isochronous; Non-iso, non-isochronous) and tempo (120 and 240 bpm).

		Arousal		Valence	

		120 bpm	240 bpm	120 bpm	240 bpm
Iso	Mean	51.27	56.36	50.96	58.45
	*SD*	19.07	22.71	19.03	18.77
Non-iso	Mean	52.88	56.28	54.25	53.80
	*SD*	14.15	16.13	12.59	15.22

*No significant differences were found across experimental conditions.*

## Discussion

In this study, we aimed at investigating whether a regular rhythmic auditory stimulation, by allowing anticipation in time, could promote forward body projections in the peri-personal space as compared to an irregular, non-isochronous rhythm. Using an approach/avoidance task in a visual virtual environment, we found that participants provided faster forward-approach (but not backward-avoidance) responses following an isochronous (but not non-isochronous) auditory stimulation. Importantly, temporal regularities promoted forward projections of body movements no matter the tempo of the auditory stimuli, thus suggesting a generalizable effect relying on the isochronous nature of the stimulation rather than on its preferred spontaneous frequency (MacDougall and Moore, 2005; Zalta et al., 2020).

Research on temporal dynamics has shown that a constant duration of intervals delineating an isochronous (rhythmic) stimulation promotes an accurate prediction about when event is likely to occur, and therefore allows attention resources to be oriented toward that moment in time (Jones and Boltz, 1989; Large and Jones, 1999; Jones et al., 2002). Such temporal predictability of isochronous sequences has been shown to consistently improve sensorimotor processing of events occurring in phase with the rhythm, thus enhancing not only perceptual sensitivity (Morillon et al., 2014) and target detection speed (Bolger et al., 2014), but also working- (Fanuel et al., 2018, 2020; Plancher et al., 2018) and long-term memory (Thavabalasingam et al., 2016) processes. Here, we considered the tight time–space relationship, according to which temporal concepts are usually prescribed to spatial locations through a past-behind and future-ahead mapping (Lakoff and Johnson, 1980; Núñez and Cooperrider, 2013). As humans, we tend to conceptualize time along the sagittal space (Bender and Beller, 2014), thus representing the past as “back” and the future as “forward” (Núñez and Cooperrider, 2013). Accordingly, many studies have shown that motor responses to past- and future-related information are significantly faster when the response direction is compatible with such sagittal mental time line (Sell and Kaschak, 2011; Rinaldi et al., 2016, 2018; Walker et al., 2017). Our findings suggest that a close link exists between temporal dynamics triggered by regular rhythms and body projections in space over such sagittal representation.

In particular, by showing for the first time that regular auditory stimulations are related to forward, but not backward body movements, our results support the idea that cognition is rooted in the interactions between the body and the environment and projected onto a temporal dimension (see Barsalou, 2008; Versace et al., 2014). From a more speculative perspective, our findings would suggest that isochronous-related benefits on perception and cognition could be based on, or at least intrinsically related to the concept of projections in space. Indeed, temporal regularities might promote the sensorimotor anticipations in space likely to benefit perceptual and cognitive processes (Barsalou, 2008; Pezzulo, 2011; Koziol et al., 2012).

In order to provide an ecological, realistic, and immersive virtual environment likely to promote participants body projections in space, we decided to employ the VAAST task (Rougier et al., 2018; see also Cereghetti et al., 2021 for an adaptation in another sensory modality). However, it is worth mentioning that the nature of the paradigm itself could suggest possible alternative interpretations of results. First, the procedure originally relied on verbal emotional stimuli and showed that participants’ responses were faster in approaching positive and avoiding negative stimuli (i.e., approach/avoidance compatibility effect, Solarz, 1960). In our study, we were interested in the impact of the temporal regularities on the time taken to produce a motor response generating a forward or backward visual flow. One might therefore argue that the faster responses following isochronous auditory stimulation are related to a more positive emotional valence associated with temporal regularities (vs. irregularities; Rougier et al., 2018). However, the affective (i.e., arousal and valence) subjective ratings provided by our participants did not show any significant difference between isochronous and non-isochronous auditory stimulations, thus excluding an approach/avoidance compatibility effect driven by hedonic-emotional responses.

Another possible interpretation of our findings relies on the fact that participants were asked to provide responses associated to approach/avoidance body movement toward the center of the screen, represented by a metronome. As the metronome is usually associated to regular rhythmic stimulation, faster approach responses following isochronous rhythms could reflect congruency effect. Further studies employing more neutral stimuli would help to disentangle the contribution of stimuli congruency in the observed findings. However and crucially, we showed that our participants were faster in approaching the metronome after an isochronous rhythm, but not in avoiding it after a non-isochronous auditory stimulation. Therefore, our results would point to a specific effect of isochronous-driven temporal prediction over body movements in space rather than a more general priming-congruency effect.

Such isochronous-driven facilitation over forward movements can also be explained by the fact that our daily body activities in space, such as walking or running, usually follow a regular rhythm and are associated with forward body movements. Many studies have shown the deep relationship between isochronous auditory sequences and ambulation. For example, convergent evidence has shown that regular beats are able to enhance perceptual and motor timings in Parkinson’s patients, in turn improving their gait (e.g., Benoit et al., 2014; Dalla Bella et al., 2017; Koshimori and Thaut, 2018). However, in our study we observed no specific facilitation of preferred walking tempo (i.e., our 120 BPM condition; see MacDougall and Moore, 2005; Zalta et al., 2020) on participants’ performance. Furthermore, the influence of time representation (i.e., backward-past, forward-future) on whole-body movements has been well documented (e.g., Rinaldi et al., 2016). It appears therefore difficult to disentangle the contribution of the motor (i.e., ambulation) and time representation (i.e., backward-past and forward-future) components in the observed findings. An interesting perspective would be to investigate, in future research, cultures in which the time representation is inverted, with a timeline representing past forward and future backward (Núñez and Sweetser, 2006; Boroditsky et al., 2011). If our results could be entirely explained by the motor-ambulation component, then this should act independently from the time–space representation, and we should observe in these cultures the same effect as the one observed in the present work. Otherwise, if the motor component works intertwined with time–space representation, then the isochronous facilitation over forward body movements should disappear or be significantly reduced.

In sum, this study shows for the first time that a tight link exists between isochronous auditory stimulations, likely to promote the anticipation of events in time, and forward-body projections in the peri-personal space. Taken together, our findings support the idea that temporal predictions driven by a regular auditory stimulation are grounded in a perception-action system integrating temporal and spatial information.

## Data Availability Statement

The raw data supporting the conclusions of this article will be made available by the authors, without undue reservation.

## Ethics Statement

Ethical review and approval were not required for the study on human participants in accordance with the local legislation and institutional requirements. The patients/participants provided their written informed consent to participate in this study.

## Author Contributions

LF, RV, and CV made substantial contributions to the conception and design of the work and the acquisition, analysis, and interpretation of data for the work. LF, RV, and GP drafted the work and revised it critically for important intellectual content. LF, RV, CV, and GP gave final approval of the version to be published and agreed to be accountable for all aspects of the work in ensuring that questions related to the accuracy and integrity of any part of the work are appropriately investigated and resolved. All authors contributed to the article and approved the submitted version.

## Conflict of Interest

The authors declare that the research was conducted in the absence of any commercial or financial relationships that could be construed as a potential conflict of interest.

## Publisher’s Note

All claims expressed in this article are solely those of the authors and do not necessarily represent those of their affiliated organizations, or those of the publisher, the editors and the reviewers. Any product that may be evaluated in this article, or claim that may be made by its manufacturer, is not guaranteed or endorsed by the publisher.
